# Reliable P wave detection in pathological ECG signals

**DOI:** 10.1038/s41598-022-10656-4

**Published:** 2022-04-21

**Authors:** Lucie Saclova, Andrea Nemcova, Radovan Smisek, Lukas Smital, Martin Vitek, Marina Ronzhina

**Affiliations:** 1 Department of Biomedical Engineering, Faculty of Electrical Engineering and Communication, Brno University of Technology, Technická 12, 616 00 Brno, Czech Republic; 2 Institute of Scientific Instruments, The Czech Academy of Sciences, Královopolská 147, 612 64 Brno, Czech Republic; 3 Department of Technical Studies, College of Polytechnics Jihlava, Tolstého 16, 586 01 Jihlava, Czech Republic

**Keywords:** Electrocardiography - EKG, Biomedical engineering

## Abstract

Accurate automated detection of P waves in ECG allows to provide fast correct diagnosis of various cardiac arrhythmias and select suitable strategy for patients’ treatment. However, P waves detection is a still challenging task, especially in long-term ECGs with manifested cardiac pathologies. Software tools used in medical practice usually fail to detect P waves under pathological conditions. Most of recently published approaches have not been tested on such the signals at all. Here we introduce a novel method for accurate and reliable P wave detection, which is success in both normal and pathological cases. Our method uses phasor transform of ECG and innovative decision rules in order to improve P waves detection in pathological signals. The rules are based on a deep knowledge of heart manifestation during various arrhythmias, such as atrial fibrillation, premature ventricular contraction, etc. By involving the rules into the decision process, we are able to find the P wave in the correct location or, alternatively, not to search for it at all. In contrast to another studies, we use three, highly variable annotated ECG databases, which contain both normal and pathological records, to objectively validate our algorithm. The results for physiological records are Se = 98.56% and PP = 99.82% for MIT-BIH Arrhythmia Database (MITDP, with MITDB P-Wave Annotations) and Se = 99.23% and PP = 99.12% for QT database. These results are comparable with other published methods. For pathological signals, the proposed method reaches Se = 96.40% and PP = 91.56% for MITDB and Se = 93.07% and PP = 88.60% for Brno University of Technology ECG Signal Database with Annotations of P wave (BUT PDB). In these signals, the proposed detector greatly outperforms other methods and, thus, represents a huge step towards effective use of fully automated ECG analysis in a real medical practice.

## Introduction

Among various examination techniques, electrocardiography (ECG) is still a highly valuable tool used for the diagnosis of many cardiovascular disorders. Cardiovascular diseases are currently the most common cause of death worldwide^1^. ECG reflects the electrical activity of the heart and provides a huge amount of information about heart function^2^. In order to diagnose a person based on ECG, cardiologists use automatic diagnostic algorithms, particularly in the case of long-term monitoring (e.g. several types of ECG holter monitor, event monitor, Apple Watch 6, Bittium, Faros etc.)^3^. In medical practice, there is many commercial software solutions used for automatic analysis of long-term ECG^4–8^. However, none of these software can reliably evaluate ECG records with no further cardiologist check-up required. Therefore, there is a strong need to develop new, more accurate, and robust methods for processing and analysing ECG records.

The fundamental steps towards identification of pathology in an ECG are automatic detection of the QRS complex, P wave and T wave^9^. The P wave reflects atrial depolarization (activation), QRS complex represents the depolarization of ventricles and T wave their repolarization. Detection of the P wave is the most complicated part of the process, and it is still not solved problem^10^. P waves detection is more difficult than the detection of other ECG components due to following reasons: (a) P waves have a low voltage, resulting in a low signal-to-noise ratio (SNR); (b) P waves have no exclusive time and frequency characteristics; (c) P waves have high interpatient variability; (d) in the case of atrioventricular (AV) dissociations, P waves do not respect normal time ordering of an ECG sequence and, thus, can be missing or redundant); (e) during tachycardia, P waves can be hidden within the T waves^3^; (f) during atrial fibrillation (AFIB) and atrial flutter (AFL), P waves are missing or replaced by so called f-waves or F-waves, respectively; (g) in the case of ventricular ectopy, P waves are usually not present at all.

The information about P waves are important to diagnose many types of arrhythmias. Particularly, the information about P waves positions can be used to diagnose AV block of the 1st, 2nd and 3rd degree. It is a key point for differentiation between supraventricular and ventricular tachycardias and for identification of junctional and ventricular ectopic beat or rhythm, atrial fibrillation and flutter. Changes in P wave shape (e.g. peaked, notched, inverted or enlarged P) may indicate atrial pathologies, such as atrial hypertrophy or enlargement and others. It may further correspond with the retrograde conduction from the AV node to the atria during junctional rhythm or traveling pacemaker^1,2^. The results of automatic P waves detection allow to gain more information from the ECG record and, consequently, simplify daily cardiologist work. Early detection of P waves and, subsequently, an illness can decrease risk of patients’ mortality^11^.

The most significant drawback of the commonly used detection algorithms (from the literature as well as the software used in clinical practice) is in assuming that the P wave is followed by the QRS complex. This is valid for normal cardiac rhythm and may not be true for pathological ones. The above algorithms search for the P wave in area before the QRS complex and detect the maximum of P wave within this area using different methods, such as adaptive thresholding^12,13^, wavelet transform^14,15^, specific P wave template and further correlation^16^, Kalman filtering^17^, moving average^13^, support vector machine^18,19^, Prony’s method^20^, the hidden Markov models^21^, neural network^22^, phasor transform (PT)^23–26^, dynamic programming^27^, combinations of several detection algorithms^28^, differential evolution^29^, etc. These approaches deliver good results when testing on ECG with normal cardiac rhythm and perform poorly in case of ECG with pathological manifestations. For example, when applying on ECG with premature ventricular contraction (PVC), the Portet’s’ algorithm^3^ achieved sensitivity (Se) = 70.37% and positive predictivity (PP) = 59.41% and the Laguna’s algorithm^30^ achieved Se = 76.14% and PP = 55.87%. Most of the published methods, however, have not been tested on pathological records at all.

In our previous work^31^, we presented the original method for P wave detection and tested it on the signals from MIT-BIH Arrhythmia Database (MITDB) using the P waves annotations from MIT-BIH Arrhythmia Database P-Wave Annotations (MIT PDB)^24,32^. Besides the normal ECG, the database contains three types of pathological records, namely ECG with PVC, AV block II degree (AVB II), and junctional rhythm. For this database, our previous algorithm achieved promising results with overall Se = 96.40% and PP = 91.56%. In order to validate the algorithm objectively on highly variable data, we created new publicly available ECG database with 23 types of pathologies and annotated P waves, which was published as Brno University of Technology ECG Signal Database with Annotations of P Wave (BUT PDB)^33^. On this database, our previous detection algorithm performed insufficiently (overall Se = 78.13%, PP = 79.67%), which motivated us to create an improved robust version of the P wave detector.

In this paper, we introduce an improved method for P wave detection in ECG. According to the results (see below), the method significantly reduces the limitations of our previous detectors^26,31^ and outperforms other recent detectors. The proposed detector consists of a phasor transform (PT) of ECG, adaptive area demarcation for P wave searching and clear decision rules that improve P wave detection, especially in pathological signals. The innovative rules are based on deep knowledge of heart manifestation during both physiological and pathological conditions. The decision process starts with the detection of the relevant pathologies in analyzed segment and selection of suitable detection criteria and ends with the acceptance of previously detected P wave candidate as true or not. As a result, we search for the P wave in the correct location or, alternatively, do not search for it at all. Unique criteria for demarcation of ECG areas used for P wave searching ensure accurate detection in data from patients with AFIB, various types of PVC, AVB II, bundle branch blocks, etc. Another benefit of our study is in testing the algorithms (newly proposed as well as its previous versions) on three different, highly variable ECG databases, which is not common in this field. Our comprehensive study replies on the need of accurate, fully automated systems for ECG analysis based on recent knowledge about heart functioning under different conditions and respecting the principles of experts’ rule-based decision making. The outputs of the study contribute to early, effective treatment of the patients by increasing the diagnostics profits of routinely used ECG.

## Methods

**Figure 1:**
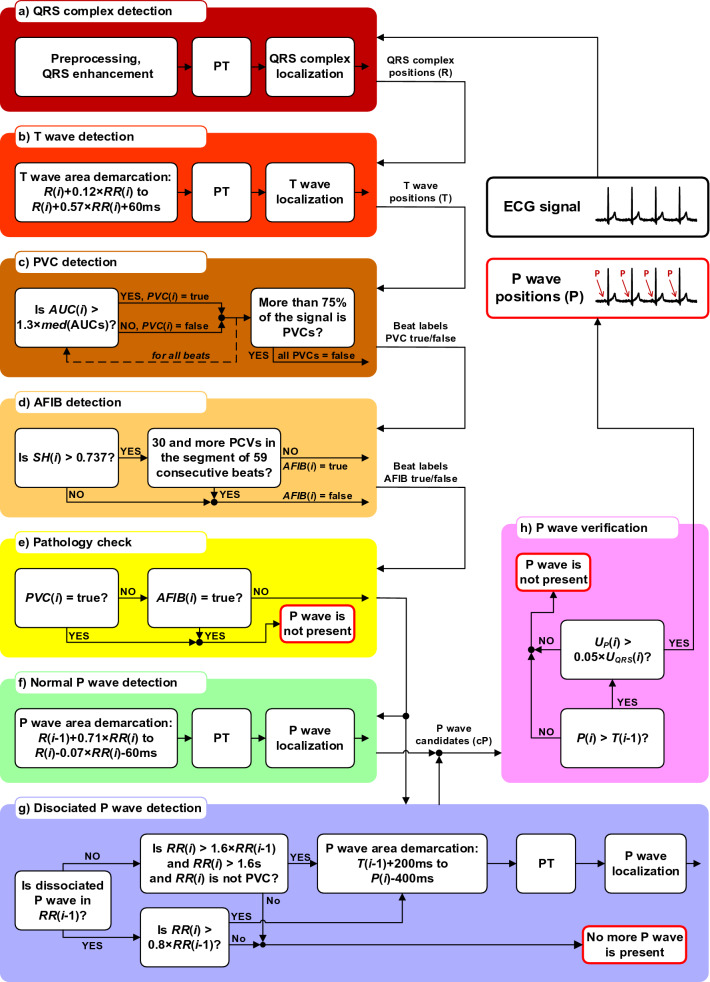
The overall process of P wave detection: (**a**) QRS complex detection using Phasor transform, (**b**) T wave detection based on the QRS complexes positions, (**c**) detection of PVC using morphological feature and correctness check, (**d**) detection of AFIB using Shannon entropy, (**e**) check of PVC or AFIB presence in ECG, (**f**) basic detection of P waves in physiological heart beats based on the QRS complexes positions, (**g**) detection of dissociated P waves within special demarcation area, (**h**) unification of P waves positions, check of P wave amplitude and verification of P wave positions correctness.

The entire algorithm for P wave detection consists of eight parts: (a) QRS complex detection, (b) T wave detection, (c) PVC detection, (d) AFIB detection, (e) pathology check, (f) normal P wave detection, (g) dissociated P wave detection, and (h) P wave verification. The complete architecture of P wave detection algorithm is demonstrated by the block diagram in Fig. 1. Each block is described in detail below. The integration of methods for AFIB and PVC detection and several novel decision rules to P wave detection algorithm is an important innovation of the proposed method.

Values and constants used during P wave detection were determined according to the knowledge of cardiac activity (definition of the area for searching for P wave, T wave, PVC, check of PVC and AFIB detection, etc.) and the properties of PT (value of Rv).

**Figure 2:**
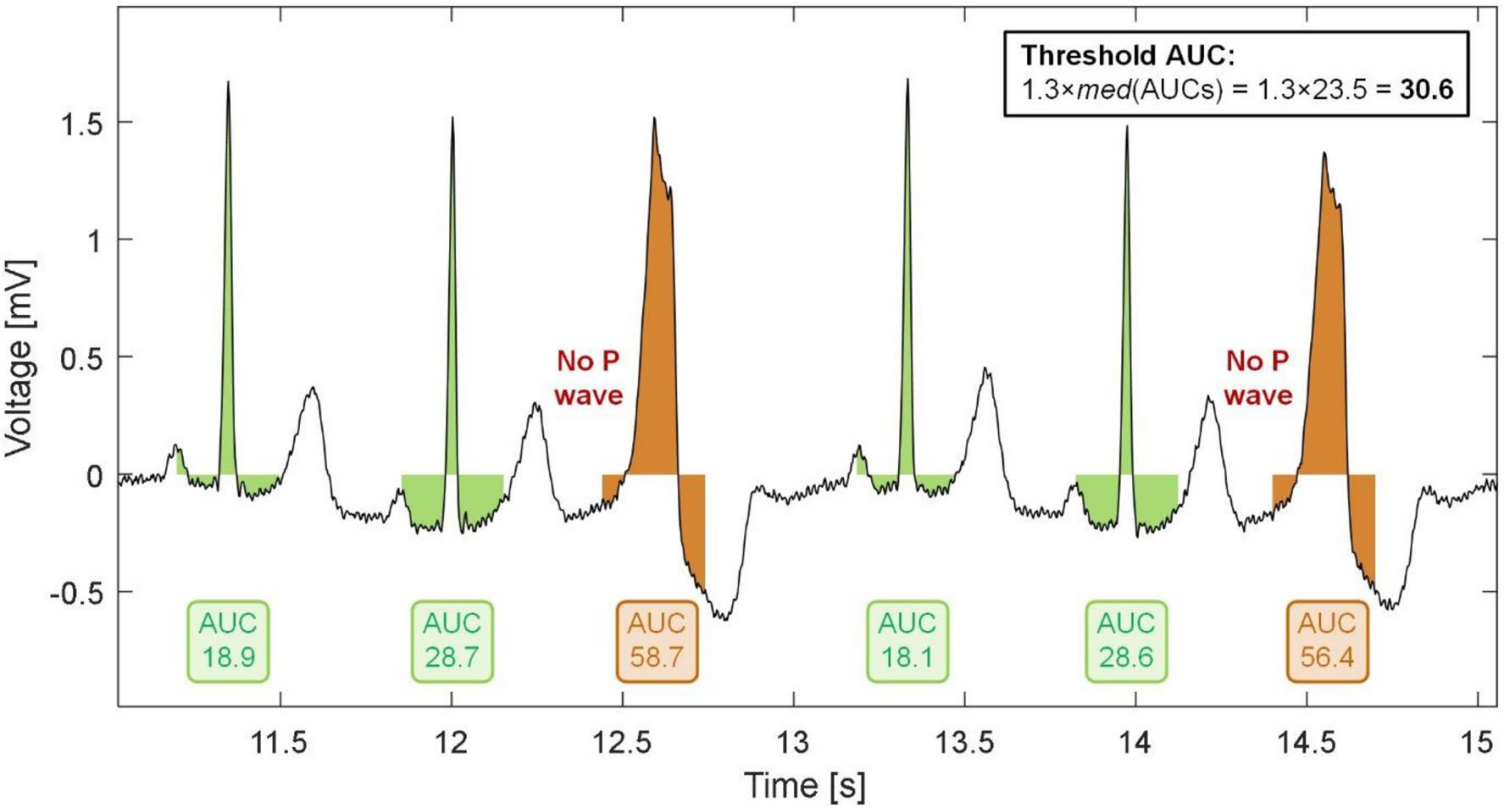
The illustration of PVC detection. The PVC is detected by thresholding the area under QRS complex (AUC) calculated in the ECG segment demarcated from *R*(*i*) − 150 ms to *R*(*i*) + 150 ms, where *R*(*i*) is the position of current QRS (R wave).
  In the next step, the number of beats labelled by algorithm as PVC is calculated. If more than 75% of all beats in ECG are labelled as PVCs, the PVC detection results are considered as a mistake. Instead, morphological changes of the beats are considered to be a sign of right or left bundle branch blocks, which are known to have similar ECG manifestations as PVC. As a result, each beat is assigned as normal or PVC, which is used in further steps.
- *AFIB detection*
  During AFIB, P waves are not present in ECG^39^ and, thus, common P wave detection algorithms produce many false positive detections. To eliminate this problem, we supplemented our algorithm by checking of the AFIB presence in current beat *R*(*i*). If the beat is marked as AFIB, the algorithm does not search for the P wave at all.
  The pilot version of AFIB detection method was published in^40^. Here, we introduce the modified approach. It is based on the representation of heart rate dynamics via so called symbolic dynamics (symbols and words) and Shannon entropy (SH). First, the heart rate sequence (*hr*(*i*)) is calculated from the RR intervals (*RR*(*i*)) and transformed into the symbol sequence (*Sy*(*i*))^41^. The 3-symbol template is then used to examine the entropic properties of *Sy*(*i*) and another 3-symbol template is used to obtain the transformed sequence of words (*wv*(*i*)). The template length was set on only 3 samples to ensure low computational demand of the sequence analysis. Second, *SH*(*i*) is computed from the segment of 59 consecutive word elements (beats) selected as *wv*(*i-29*) to *wv*(*i* + *29*). Finally, the beats with *SH*(*i*) higher than 0.737 (selected empirically) are marked as AFIB, since during AFIB, *RR* intervals are highly variable resulting in a large *SH*^41,42^.
  In Fig. 3, the process of AFIB detection is illustrated. Upper graph shows the lengths of RR intervals and lower graph shows the corresponding *SH* values and the decision threshold for AFIB detection. It is obvious from the figure, that the increased *SH* values (above the threshold) correlate with the presence of AFIB in ECG (according to the ground truth annotations available in a database).

- *QRS complex detection*
  Firstly, QRS complex detection is provided. The raw signal is preprocessed^34,35^ and filtered by a bandpass FIR filter with Hamming window and passband of 12 to 19 Hz^36^ in order to enhance the QRS complexes and suppress P and T waves. After filtration, Phasor transform (PT) is applied on the signal. PT enhances variations of the signal’s components (such as P waves, T waves and QRS complexes) and makes the detection of these components easier^23^. PT transforms each sample of the signal into a complex value preserving the signal information. The constant value *R*_*V*_ is considered as a real part of the phasor signal, while the value *x*(*n*) of the original ECG sample is considered as an imaginary component: $y\left( n \right) = R_{V} + jx\left( n \right)$. *R*_*V*_ is set on the value within the interval 0–1, which indicates the ‘degree’ of the waves enhancement in ECG. For QRS detection, *R*_*V*_ = 0.001 is used. The phase (phasor) signal $PT\left( n \right)$ is then computed as $PT\left( n \right) = tan^{ - 1} \left( {\frac{x\left( n \right)}{{R_{V} }}} \right).$
  In signal $PT\left( n \right)$, maxima are detected in sliding window (300 ms long) and compared to an adaptive threshold established as a double of standard deviation calculated in 2 s moving window. The positions of maxima, which are higher than the threshold, are considered as the positions of QRSs (R waves). If the current RR interval *RR*(*i*) is 1.75 times longer than the previous one *RR*(*i* − 1), backward searching with a new threshold established as 30% of the last detected QRS amplitude is additionally applied to add possible missing detections. More detailed information about QRS detection via PT can be found in our previous work^31^. The output of this step—QRS positions—is then used for demarcation of areas for P and T waves searching and for detection of PVC and AFIB.
- *T wave detection*
  The searching area for T wave detection is determined using the position of the corresponding QRS complex *R*(*i*) as *R*(*i*) + 0.12 × *RR*(*i*) to *R*(*i*) + 0.57 × *RR*(*i*) + 60 ms. In this area, the ECG is transformed using the PT in the same way as in the case of QRS detection, but with *R*_*V*_ = 0.1. The maximum of the phase signal *PT*(*n*) within the demarcated segment is considered as the position of the T wave. We already used a similar method^31^, but here we introduce different way for demarcating of the searching area. The T waves positions are further used to identify the area for P wave searching.
- *PVC detection*
  Recognition of PVC is a very important step for demarcating the area where P wave may occur. If the current beat *R*(*i*) is marked as PVC, the P wave may not be searched before the QRS complex, because the P wave is not present at all. The proposed PVC detection method is effective, with low computational cost. It is based on only one simple feature extracted from the QRS, namely the area under the QRS (*AUC*(*i*)) calculated from the segment demarcated using current R wave position as *R*(*i*) − 150 ms to *R*(*i*) + 150 ms. Before *AUC* calculation, the signal is filtered using a high-pass Lynn’s filter with a cut-off frequency of 0.67 Hz to eliminate the baseline wandering. The current beat is then considered as PVC, if its *AUC*(*i*) is 1.3 times larger than a median *AUC* calculated from all previous beats. An example of PVC detection procedure is shown in Fig. 2. In previous studies, we used multi-feature approach for PVC detection^24,37,38^, where we combined *AUC* with other features. Here, we achieved promising results by using *AUC* only (see below).

**Figure 3:**
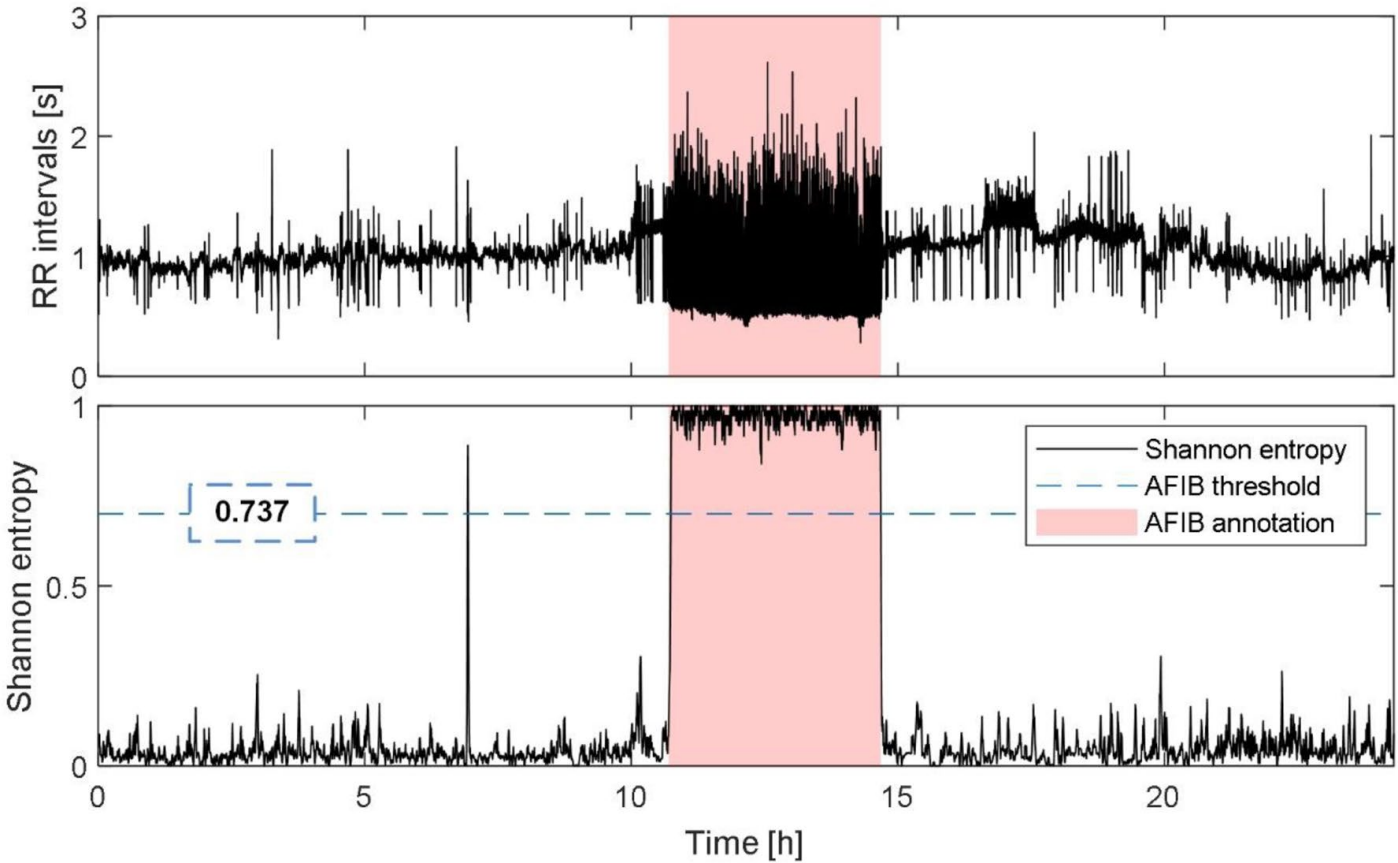
The illustration of AFIB detection. Top: Length of RR intervals. Bottom: Corresponding Shannon entropy with decision threshold (blue line) and ground truth AFIB annotations (red block). Shannon entropy values higher than the threshold (about 0.9–1 in this particular case) correspond with ECG segment, where AFIB manifestations are present (according to the annotations available from the database).
  In ECG with detected AFIB, the PVCs number is then calculated within the segments of the 59 consecutive beats. If more than 30 PVCs (50% of total beats) is present in the segment, than increased *SH* values seem to be due to the PVCs, which are ‘surrounded’ by RR intervals of specific lengths (shortened and extended for RR before and after the PVC, respectively) different from the lengths of RR intervals surrounding the normal beats. In this case, the current beat *R*(*i*) is not considered as AFIB. As a result, each beat is labeled as normal or AFIB and this information is involved into further analysis.
- *Pathology check*
  In this stage, the algorithm checks, whether the pathologies from the steps (c) and (d) were detected in the current beat, and decides, whether P wave detection process continues or not. Particularly, if the beat is marked as AFIB, the P wave detection in this beat is terminated (see above). In the beats with no AFIB, the presence of detected PVC is checked. If the beat is marked as PVC, then the detection process is terminated (see above). In the opposite case, the algorithm continues to the step f).
- *Normal P wave detection*
  If the currently analyzed beat is not labeled as PVC nor AFIB, the segment for P wave searching is selected from ECG as *R*(*i* − 1) + 0.71 × *RR*(*i*) to *R*(*i*)-0.07 × *RR*(*i*)*-*60 ms and transformed by the PT with *R*_*V*_ = 0.05. The maximum peak from the calculated phase signal is then considered as a P wave candidate (cP). In Fig. 4, an example of P wave searching is shown. For the first beat of signal, the segment for P wave searching is set as *R*(*i*)-300 ms to *R*(*i*)-80 ms.

**Figure 4:**
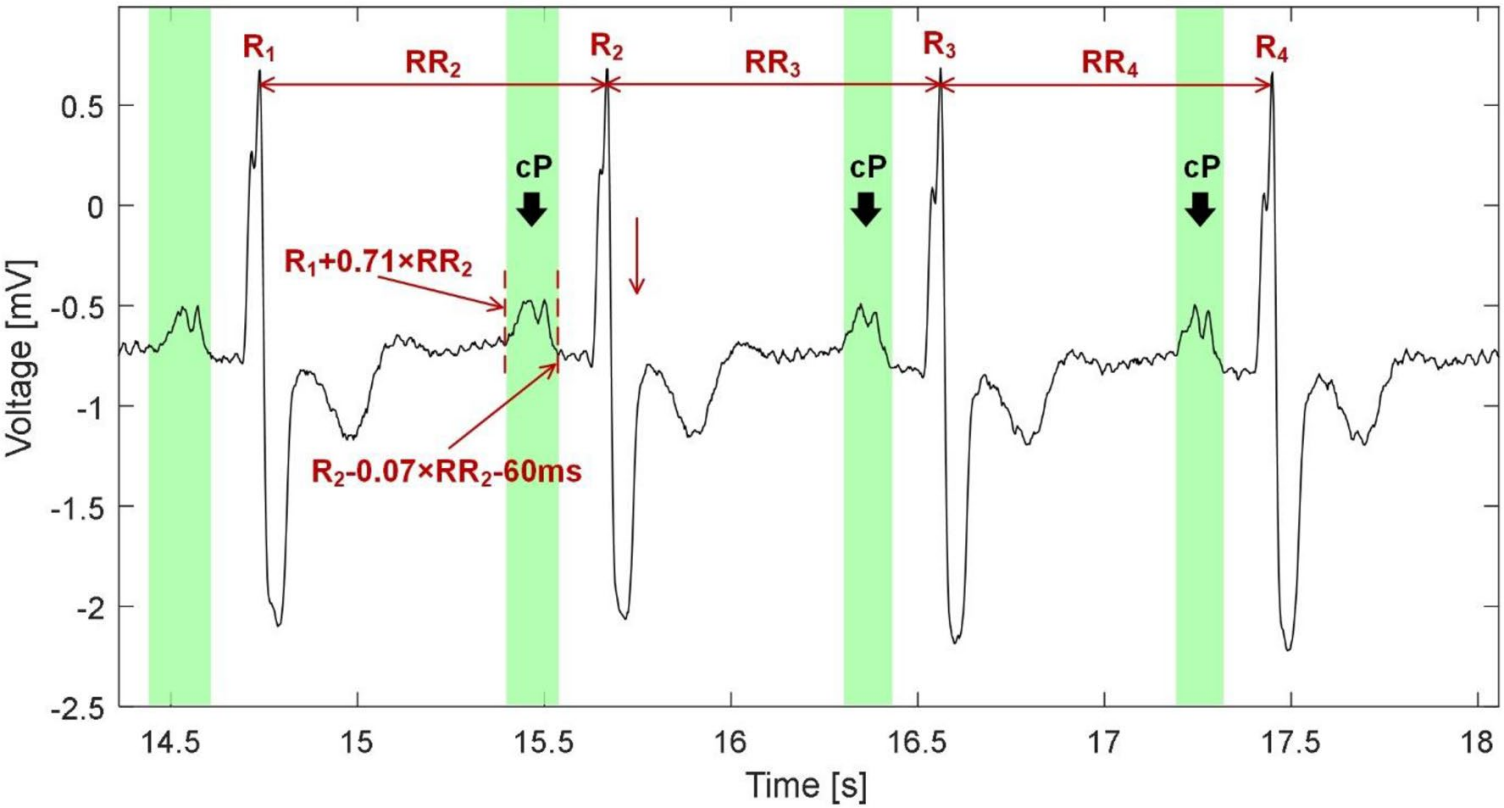
Normal P waves detection. Searching areas (green blocks) are demarcated as *R*(*i* − 1) + 0.71 × *RR*(*i*) to *R*(*i*)-0.07 × *RR*(*i*)*-*60 ms and the P wave candidates (cP) are found as the maximum peaks in the areas processed by a phasor transform (not shown here).
- *Dissociated P wave detection*
  Dissociated P waves can be usually found in ECG of patients with AVB II. To detect these waves carefully, we proposed simple criteria. First, it is checked, whether there is a dissociated P wave in the previous RR interval (*RR*(*i* − 1)). If not, then three further criteria are checked: (1) *RR*(*i*) > 1.6 × *RR*(*i* − 1), (2) *RR*(*i)* > 1.6 s (3) current beat *R*(*i*) is not PVC. If the dissociated P wave was found in the previous interval, then one criterion is checked: *RR*(*i*) > 0.8 × *RR*(*i* − 1). In both cases, if the criteria are met, the dissociated P wave may be present in the current beat and, thus, the position of this wave is further detected. If the above criteria are not met, the dissociated P wave is not present in the beat and the detection procedure is terminated. The dissociated P wave is localized in the segment demarcated as *T*(*i* − 1) + 200 ms to *P*(*i*)*-*400 ms. The segment is transformed by the PT in the same way as in step f) and the position of P wave candidate cP is found by detecting the maximum peak within the segment. In Fig. 5, the detection of dissociated P waves is illustrated.

**Figure 5:**
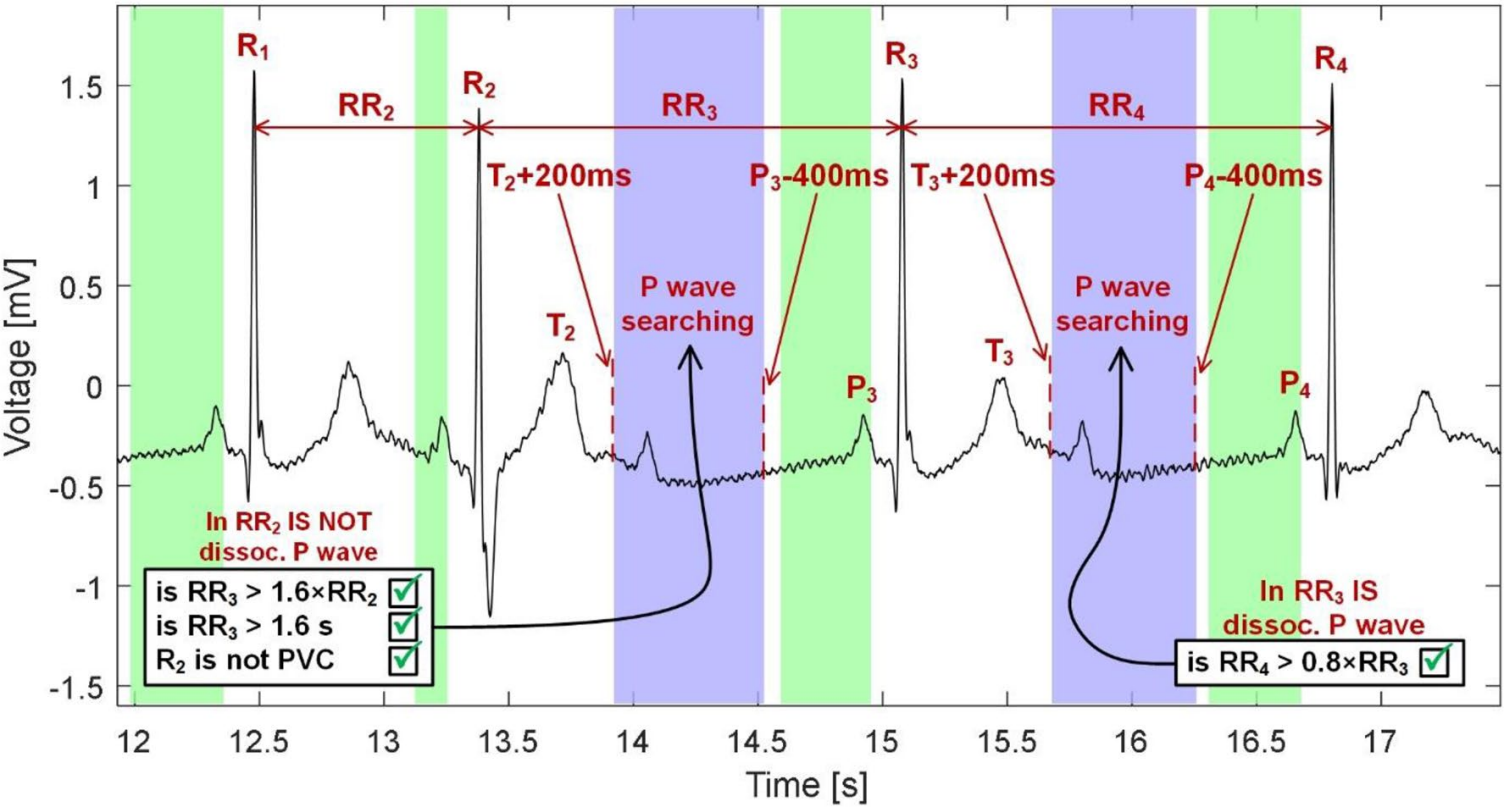
Demarcation of segments for P wave searching in the case of possible dissociated P waves presence.
- *P wave verification*
  In the last step, the P waves candidates are validated. First, the voltage level of the candidate *cP*(*i*) is assessed by the criterion: *U*_*P*_(*i*) > 0.05 × *U*_*QRS*_(*i*). If the criterion is not met, the P wave is probably not present in the current beat, which may be in case of nodal origin of the beat/rhythm or idioventricular rhythm. Second, the position of the candidate *cP*(*i*) is verified to be ensure, that the candidate is not a part of the previous T wave, but the true P wave of the current beat. Corresponding criterion is *cP*(*i*) > *T*(*i* − 1), where *cP*(*i*) is the position of current P wave candidate and *T*(*i* − 1) is the position of the previous T wave. If this criterion is not met, this P wave candidate is excluded from the analysis, as it likely represents the T wave from previous beat instead of P wave of current beat. Consequently, the true P wave is most probably absent in the current beat or is hidden in the previous QRS complex or previous T wave, such it happens in case of supraventricular tachyarrhythmia, sinus tachyarrhythmia or atrial premature beat. If the above criterion is met, the candidate position *cP*(*i*) is considered as the position of P wave.

### Testing databases

For testing the proposed algorithm, physiological as well as pathological ECG records with P waves annotated by the experts were needed. There are only three publicly available databases, which contain correct manual annotations of P waves. All the databases can be found on Physionet^32^. In all databases, the first lead was used for algorithm testing.

The first database is a part of *MIT-BIH Arrhythmia Database* (MITDB)^32,43^ with the P wave annotations published by our team for selected ECGs under the name *MIT-BIH Arrhythmia Database P-Wave Annotations* (MIT PDB)^24^. For this database, the P wave annotations were also published by Elgendi et al.^44^. However, these annotations contain many mistakes and, thus, they are not suitable for reliable testing of detection algorithms. The MITDB dataset is widely used database for evaluation of QRS detectors and is the most cited ECG database at all^45^. It contains both physiological and pathological ECG records sampled with frequency 360 Hz. For our study, we selected 12 physiological and pathological signals with P wave annotations available. Particularly, selected records no. 106, 119, 214, and 223 include PVCs (various types of ventricular arrhythmias—ventricular bigeminy (B), ventricular trigeminy (T), and idioventricular rhythm (IVR). Records no. 207 and 222 include nodal rhythm (NOD) and record no. 231 includes AVB II. Records no. 100, 101, 103, 117, and 122 do not include any significant pathology. Therefore, these records represent normal signals, which will be used to verify the performance of the algorithm under physiological conditions. Altogether, the database contains 2281 P waves.

The second database is the *QT database* (QTDB)^32,46^. It consists of 105 15-min-long two-channel ECG records sampled at 250 Hz. In this study, the first channel was used. For all records and beats, the automatically found reference positions of QRS complexes are available. For some beats, the QTDB includes manual annotations of P wave peak, P wave onset, P wave offset, QRS complex onset, QRS complex offset, T wave peak and the T wave offset. All annotations are available for at least 30 beats per record in 79 out of the 105 recordings^46^. The performance of the proposed algorithms for P waves and T waves detection was tested against the manually annotated part of the QTDB (altogether 3622 beats), which mainly represents the physiological signals.

The third database is *Brno University of Technology ECG Signal Database with Annotations of P Wave* (BUT PDB) recently published by our team^33^. It consists of 50 2-min long, two-channel ECG records with 23 different types of pathologies and manually annotated P waves. The ECGs were selected from 3 existing databases of ECG signals—MITDB, MIT-BIH Supraventricular Arrhythmia Database (MITSVA) and Long Term AF Database (LTAF)^46^. The sampling frequency is 360 Hz for signals from MITDB and MITSVA and 128 Hz for signals from LTAF. Each record from BUT PDB contains annotation of dominant diagnosis (pathology) and types of QRS complexes (taken over from the original databases). Available information about pathologies was manually checked. Since the original annotations were found correct, the labels were taken over from the original databases. The missing annotations (all signals from MITSVA) were further supplemented by ECG experts. The BUT PDB consists of 7638 QRS complexes. For 2209 QRSs, there are not P wave presented (the case of atrial fibrillation, ventricular beats or nodal rhythm). On the contrary, 141 P waves are not corresponded with QRS complexes (mainly the case of the 2^nd^ or 3^nd^ degree atrioventricular block and paced rhythm). Altogether, the BUT PDB includes 5429 P waves. Types of pathologies, their abbreviations and the number of signals in particular pathological groups are listed in Table 1. It should be noted, that the BUT PDB contains all known pathologies that affect P waves presence and/or positions.

**Table 1:** List of pathologies present in BUT PDB, their abbreviations (Abb.), number of heartbeats and the number and IDs of signals with the given pathology.

Abb.	Type of pathology	Number of heartbeats	Number of records	IDs of the records with the pathology
A	Atrial premature beat	142	21	01,04,05,09,16,17,18,26,28,31,32,35,38,39,40,41,42,43,46,49,50
SVTA	Supraventricular tachyarrhythmia	Included in A	3	09,11,43
AFIB	Atrial fibrillation	1079	9	07,08,44,45,46,47,48,49, 50
AFL	Atrial flutter	86	1	38
BI	1st degree atrioventricular block	Included in L(140)	1	22
BII	2nd degree atrioventricular block	*Extra 80 P wave*	2	1,13
BIII	3rd degree atrioventricular block	*Extra 61 P wave*	1	3
E	Ventricular escape beat	99	1	9
F	Fusion beat	76	7	06,10,14,19,32,35,36
J	Nodal beat	26	2	7,38
L	Left bundle branch block beat	448	4	21,22,36,41
NA	Sinus arrhythmia	129	1	24
NOD	Nodal premature beat	76	2	6,15
P	Paced rhythm	236	2	3,19
PREX	Pre-excitation	130	1	12
R	Right bundle branch block beat	717	6	01,06,13,26,33,34
V	Ventricular premature beat	547	27	02,03,05,08,10,14,20,21,22,25,26,27,28,29,
				30,31,32,33,35,36,37,39,40,41,42,45,47,50
B	Ventricular bigeminy	included in V	3	02,14,27
T	Ventricular trigeminy	included in V	2	27,29
IVR	Idioventricular rhythm	included in V	1	30
VP	Ventricular pair	included in V	1	25
VFL	Ventricular flutter	66	1	33
a	Aberrated atrial premature beat	9	1	23
N	Normal beat	3772		

## Results and discussion

Proposed detection algorithm was tested on physiological as well as pathological signals. Physiological signals are represented by the whole manually annotated part of QTDB^46^ and the records no. 100, 101, 103, 117, and 122 from MITDB^32,43^ with annotations MIT PDB^24,32^. Pathological signals include the records no. 106, 119, 207, 214, 222, 223, and 231 from the MITDB with annotations MIT PDB and all signals from our new database BUT PDB^33^.

Besides the results obtained by using of the proposed improved P wave detector, we also present the results of our previous algorithms. All the methods were tested on the same dataset. The first previously published algorithm is the basic P wave detector based on using the phasor transformation with no extra decision rules for pathological cases^26^. The second algorithm was specially designed for using under normal conditions (physiological cardiac rhythm) and during PVC or AVB II^31^. Here, we will compare the results of all three detectors in order to objectively evaluate the impact of the procedures we proposed for improvement of the previous outputs. As was mentioned above, these procedures are preliminary focused on eliminating of false positive P wave detections, which are common in case of many cardiac arrhythmias. From our results (see below), the number of false positives could be effectively reduced by involving the decision rules for accurate demarcation of the search area and information about presence of arrhythmia, such as AFIB and PVC.

### Detection of P waves in physiological conditions

First of all, we validated the efficiency of the proposed P wave detector under normal conditions by testing it on the ECGs with no pathology (see above). The results obtained by the proposed method as well as our two previously published detectors are summarized in Tables 2 and 3. For both test databases, the results of other teams are available. We included this data in the tables for comparison. In these studies, various methods were used to detect the P waves, such as PP rhythm tracking^3^, phasor transform^23^, wavelet transform^15^, correlation analysis^16^, parametric mixture Gaussian and dynamic programming^27^, and differential evolution^29^. From Tables 2 and 3, the results of newly proposed detector are comparable with our previous methods as well as methods published by other authors. High Se and PP values (about 98.5–99.8%) indicate that the proposed approach perform well under physiological conditions and does not bring many false detections of the P waves (false positives nor false negatives).

**Table 2:** The performance of the P wave detection algorithms on physiological signals from MITDB with annotations MIT PDB (Se—sensitivity; PP—positive predictivity; N/A—not available).

Sig. no	PP rhythm tracking^3^		Basic method^26^		Previous method^31^		Proposed method	
	Se [%]	PP [%]	Se [%]	PP [%]	Se [%]	PP [%]	Se [%]	PP [%]
100	N/A	N/A	100.0	99.3	99.69	99.25	95.13	99.31
101	N/A	N/A	99.84	99.79	98.93	99.39	98.45	99.95
103	N/A	N/A	46.76	41.84	98.8	100	99.81	100.00
117	N/A	N/A	100	99.93	96.48	99.93	99.93	99.93
122	N/A	N/A	52.35	34.25	98.18	100	100.00	99.96
Mean	99.57	99.83	79.79	75.02	98.42	99.71	98.59	99.82

**Table 3:** The performance of the P wave detection algorithms on physiological signals from the manually annotated part of QTDB (Se—sensitivity; PP—positive predictivity).

Method	Se [%]	PP [%]
Proposed method	99.23	99.12
Previous method^31^	99.84	99.84
Basic method^26^	99.85	99.83
Phasor transform^23^	99.28	99.75
Wavelet transform^15^	98.87	91.03
Correlation of template^16^	99.63	98.00
Parametric mixture Gaussian and dynamic programming^27^	96.13	97.70
Differential evolution^29^	98.90	98.50

### Detection of P waves in pathological conditions

In Tables 4 and 5, the results of detectors testing on signals with pathologies (see above) are shown. As can be seen in the tables, Se and PP were calculated for each signal separately and then averaged over the database. In the second columns of the tables, the pathologies prevailing in the particular record are noted. According to the detection results, the newly proposed method performs notably better than both previous approaches. In case of MITDB, however, this predominance is not as prominent as for BUT PDB (compare averaged Se and PP from Tables 4, 5). It is due to the fact that MITDB contains only a few types of pathologies, whereas BUT PDB includes highly variable data and, thus, allows to reveal the limitations of the previous algorithm^31^ on one side and to highlight the benefits of the novel method on the other side.

**Table 4:** The performance of the P wave detection algorithms on pathological signals from the MITDB with annotations MIT PDB (Se—sensitivity; PP—positive predictivity).

Sig. no	Type of pathology	Basic method^26^		Previous method^31^		Proposed method	
		Se [%]	PP [%]	Se [%]	PP [%]	Se [%]	PP [%]
106	PVC	90.98	91.83	92.77	81.09	99.37	94.75
119	PVC	99.38	99.69	97.41	98.20	98.15	97.80
207	NOD	81.54	56.58	96.18	78.49	97.47	78.85
214	PVC	98.55	99.5	99.75	95.32	99.90	94.45
222	NOD	82.28	54.17	62.13	89.87	81.96	84.32
223	PVC	94.62	83.72	98.00	83.86	99.48	92.27
231	AVB II	78.39	99.68	100.00	98.66	98.50	98.45
Mean		89.39	83.59	93.88	89.31	96.40	91.56

**Table 5:** The performance of the P wave detection algorithms on pathological signals from BUT PDB (Se—sensitivity, PP—positive predictivity, NaN—the whole signal is AFIB, no P waves are present, A—atrial premature beat, AFIB—atrial fibrillation, AFL—atrial flutter, B—ventricular bigeminy, BI—atrioventricular block 1st degree, BII—atrioventricular block 2nd degree, BIII—atrioventricular block 3rd degree, E—ventricular escape beat, F—fusion of ventricular and normal beat, IVR—idioventricular rhythm, J—nodal beat, L—left bundle branch block beat, NA—sinus arrhythmia, NOD—nodal rhythm, P—paced rhythm, PREX—pre-excitation, R—right bundle branch block beat, SVTA—supraventricular tachyarrhythmia, T—ventricular trigeminy, V—ventricular premature beat, VFL—ventricular flutter, VP—ventricular pair, a—aberrated atrial premature beat).

Signal no	Type of pathology	Basic method^26^		Previous method^31^		Proposed method	
		Se [%]	PP [%]	Se [%]	PP [%]	Se [%]	PP [%]
1	BII, R, A	73.48	97.00	99.24	98.50	99.24	99.24
2	V, B	98.77	58.82	98.77	97.56	91.36	91.16
3	BIII, V, P	53.57	25.21	80.36	33.83	92.86	36.88
4	A,V	99.13	98.28	92.17	67.09	99.13	99.13
5	A, V	100.00	99.29	85.61	90.84	99.28	98.57
6	NOD, F, R	96.92	62.38	93.85	68.54	100.00	69.15
7	AFIB, J	92.59	54.35	98.15	51.46	98.15	77.94
8	AFIB, V	NaN	NaN	NaN	NaN	NaN	NaN
9	E, A, SVTA	40.00	7.81	52.00	13.27	60.00	11.36
10	V, T, F	80.87	84.09	34.97	82.05	81.42	97.39
11	SVTA	35.36	86.49	88.40	99.38	100.00	97.84
12	PR	65.15	100.00	98.48	98.48	99.24	99.24
13	BII, R	57.86	98.78	63.57	100.00	78.57	100.00
14	V, B, F	100.00	73.10	54.17	86.67	100.00	95.36
15	J	100.00	97.26	91.55	91.55	100.00	79.78
16	A	99.19	99.19	97.58	100.00	100.00	100.00
17	A	100.00	99.42	98.25	100.00	100.00	99.42
18	A	76.12	99.03	86.57	100.00	79.85	100.00
19	P, F	90.00	32.14	97.50	28.26	85.00	26.15
20	V	98.13	89.71	95.63	95.03	85.63	99.28
21	L, V	51.88	64.84	76.88	91.79	100.00	98.77
22	BI, V	56.83	94.05	72.66	98.06	99.28	98.57
23	a	99.17	97.56	100.00	78.57	100.00	98.37
24	NA	35.66	100.00	99.22	100.00	100.00	100.00
25	V, VP	93.67	96.10	93.04	100.00	93.04	100.00
26	R, A, V	100.00	100.00	82.14	88.46	100.00	100.00
27	V, B, T	88.30	66.40	59.57	58.95	97.87	96.84
28	A, V	28.45	91.67	100.00	99.15	100.00	98.31
29	V	100.00	99.00	98.99	100.00	100.00	99.00
30	IVR, T, V	94.44	90.43	100.00	91.84	100.00	88.24
31	V, VT,A	49.30	76.09	98.59	92.72	97.18	94.52
32	V, F, VT,A	71.18	87.05	98.82	95.45	99.41	99.41
33	V, VFL,R	83.33	32.79	89.58	64.18	87.50	79.25
34	R	93.42	98.84	88.95	93.87	100.00	99.42
35	V, a, F	96.14	97.55	14.98	96.88	98.07	99.02
36	V, L, F	62.77	87.76	87.59	100.00	100.00	95.80
37	V, FIB	100.00	52.80	89.55	48.00	98.51	66.00
38	J, AFL, A	94.25	56.16	78.16	68.00	3.45	60.00
39	V, A	97.50	59.39	49.17	100.00	59.17	97.26
40	V, A	100.00	78.49	76.71	98.25	100.00	98.65
41	L, A, V	93.02	96.00	18.60	85.71	98.45	96.95
42	V, A	85.84	65.99	64.60	86.90	100.00	83.70
43	A, SVTA	76.19	97.56	66.67	98.59	98.10	97.17
44	AFIB	100.00	63.64	74.03	65.52	98.70	79.17
45	AFIB, V	81.48	70.51	64.44	86.14	97.04	92.25
46	AFIB, A	93.22	27.36	88.14	54.17	98.31	84.06
47	AFIB, A, V	97.30	19.67	45.95	24.64	0.00	100.00
48	AFIB	NaN	NaN	NaN	NaN	NaN	NaN
49	AFIB, A	37.08	31.73	17.98	11.43	98.88	70.40
50	AFIB, A, V	100.00	3.33	50.00	3.80	0.00	100.00
Mean		80.79	72.04	78.13	79.67	93.07	88.60

**Figure 6:**
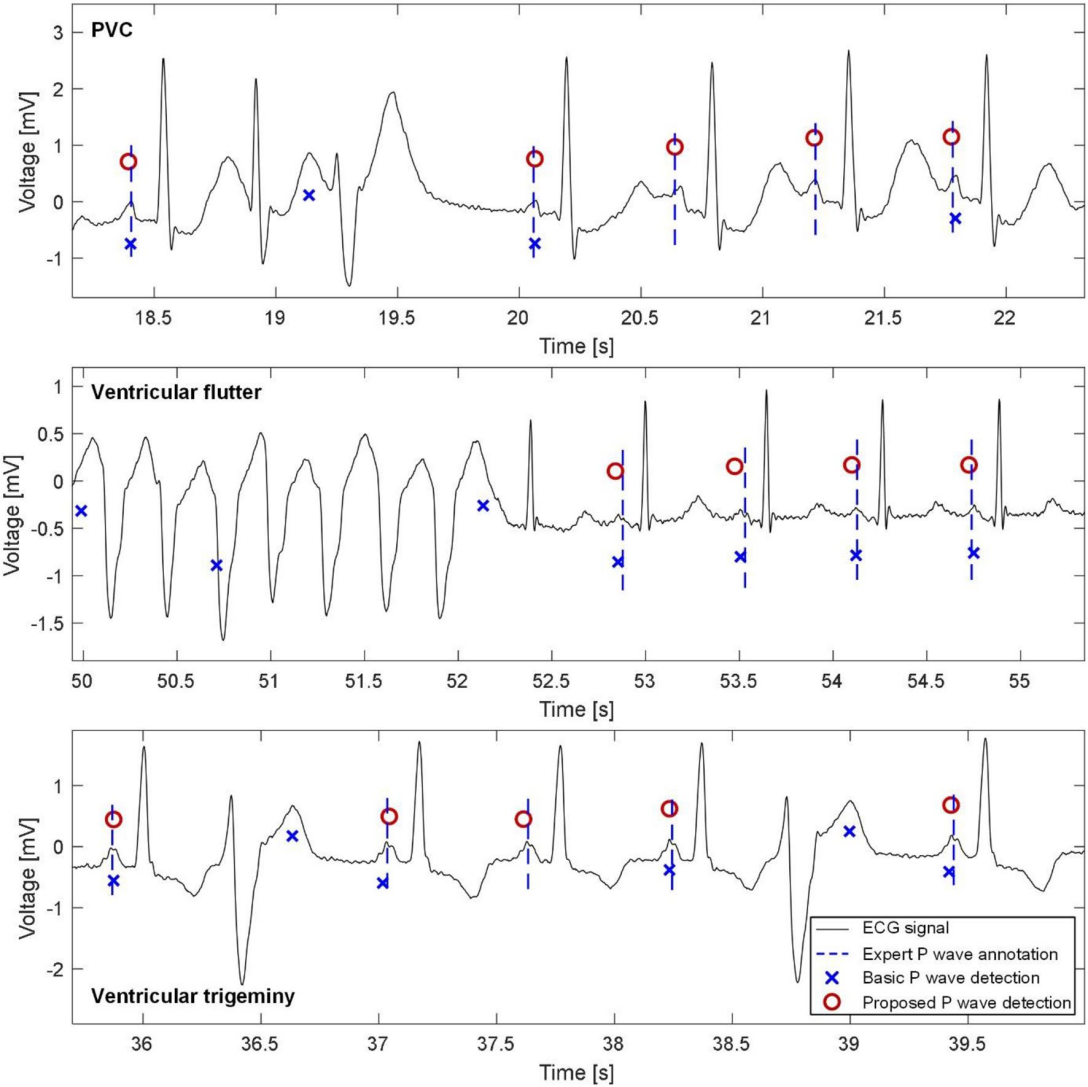
Example of P waves detection in ECGs with various pathologies. Top: Record no. 35 with single PVC. Middle: Record no. 33 with ventricular flutter episode. Bottom: Record no. 14 with ventricular trigeminy. All ECGs are from BUT PDB.

Particularly, the proposed improved algorithm achieved higher performance in most signals with all types of PVC (i.e. single PVC, bigeminy, trigeminy, ventricular pair, ventricular flutter, and fusion of normal and ventricular beat) as compared to the previous versions. The examples of P waves detection in ECG with a single PVC (record no. 35), ventricular flutter (record no. 33) and ventricular trigemini (record no. 14) are shown in Fig. 6. In all the signals, the proposed method was able to deal with a given pathology and to detect all P waves correctly. On the contrary, the basic detector failed in all cases (see false positive/negative detections in the figures).

**Figure 7:**
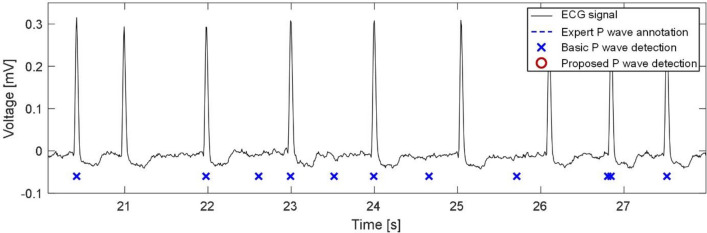
Example of P waves detection during AFIB (signal no. 48 from BUT PDB).

The next significant improvement was indicated when detecting P waves by new detector in ECGs with AFIB, which is due to special unique criteria added to the algorithm (see above). On the contrary, two previous versions are not “equipped” by the mechanisms for AFIB identification and, thus, are not able to adjust the detection process to this pathological condition. As a result, many false positive detections can be seen in output of these algorithms, as shown in Fig. 7. The ECG from the figure is entirely burdened by AFIB, which manifests in absent P waves (as was correctly recognized by the proposed detector). In a few cases, however, Se of the proposed detector was lower than that of the previous approaches (see Table 5). It can be explained by false positive detections of AFIB at the beginning or the end of the segments due to delay caused by computing SH from 59 consecutive beats.

**Figure 8:**
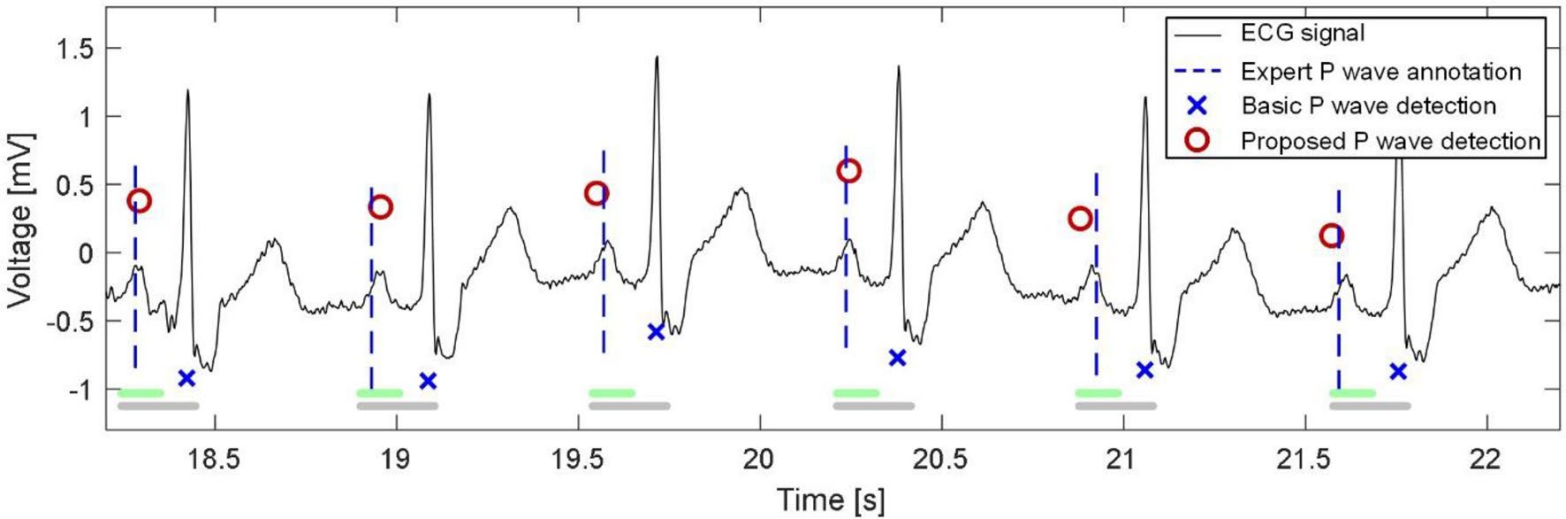
Example of P waves detection in ECG with RBBB (signal no. 34 from BUT PDB) using the search areas defined based on the previous (grey lines, basic method) and improved (green lines, the proposed method) criteria.

P waves were successfully detected in ECGs with right bundle branch block (RBBB) as well. This pathology is manifested in ECG by changed QRS complexes (wide, of higher amplitude and aberrant morphology as compared to the normal, narrow QRS). The correct detection under this condition is possible due to improved criteria for searching area. Particularly, the area was shortened by shifting its right boundary to the left on 60 ms (see section Normal P wave detection). Use of this narrow search area instead of the previously proposed wide area^31^ allows us to avoid the situations, where the QRS complexes were detected instead of the P waves (see Fig. 8).

In general, the novel algorithm reached more promising results than the previously published detectors in all pathological cases addressed in the study, including AVB II, nodal rhythm, all types of atrial and ventricular arrhythmias, bundle branch blocks, and pre-excitation.

Relatively poor results were obtained in ECGs with multiple concurrent pathologies (such as in records no. 9, 19, 33, 37 and 38 from BUT PDB). It is caused by false positive AFIB detections (and, consequently, false negative detections of P waves in corresponding ECG segments) or missed PVC detections due to highly irregular rhythm originated in overlapped manifestations of multiple arrhythmias in the same segment. Our detector was not success when testing on ECG with AVB III (record no. 3 from BUT PDB), where P waves and QRS complexes appear in ECG independently from each other.

There are only few published studies reporting the performance of P wave detectors on the available databases. Therefore, the comparison of our results with the results of other teams is rather limited and can be provided only on the manually annotated signals from MITDB. On this database, Laguna et al.^30^ achieved averaged (over all the signals with PVC, NOD and AFIB) Se = 71.13% and PP = 59.08% using the multilead detector. The PP rhythm tracking method proposed by Portet et al.^3^ reached averaged Se = 61.89% and PP = 59.00% on the same ECGs. Vitek et al.^47^ applied wavelet transformation and decision rules and detected P waves with averaged Se = 90.79% and PP = 84.56%. It is obvious, that our detector with averaged Se = 96.4% and PP = 91.56% significantly outperforms above approaches. Taking into account the results on all three databases, the proposed detector is a promising tool for analysis of ECG recorded in patients with many different arrhythmias.

## Limitations of the study

The main limitation of this study is that the proposed algorithm was not tested on ECGs with extensive noise and artefacts. In these situations, therefore, successful P wave detection cannot be guaranteed. The algorithm seems to be inaccurate when detecting P waves in ECGs with junctional rhythm and AVB III. To provide more comprehensive evaluation of detector performance, it should be tested on more ECGs. However, to the best of our knowledge, there are no other databases suitable for reliable testing of P waves detectors.

## Conclusion

This work introduces a new advanced method for P wave detection in ECGs based on a combination of simple phasor transform of the signal and innovative set of decision rules. Involving of unique criteria into the algorithm significantly improved P wave detection during pathological events, which is still a challenging task. The criteria are based on deep knowledge of heart manifestations during both normal and pathological conditions, such as AFIB, PVC, RBBB, etc. The main benefit of the criteria is in accurate definition of searching areas based on information about pathologies present in the current segment. As a result, the algorithm adjusts its parameters in order to eliminate false positive and false negative P waves detections.

Under normal conditions, the algorithm achieves similar results as previously published methods with Se = 98.56% and PP = 99.82% for ECGs from MIT PDB, and Se = 99.23% and PP = 99.12% for ECGs from QTDB. In ECGs with pathological manifestations our algorithm prominently outperforms other approaches, as follows from the comprehensive testing on highly variable datasets from MIT PDB (Se = 96.40%, PP = 91.56%) and BUT PDB (Se = 93.07%, PP = 88.60%). It should be noted, that the latter contains all the known pathologies affecting P waves presence and positions in ECG.

By accurate automatic detection of P waves in ECGs, our method has a potential to improve the diagnostic yield of routine ECG examination and to simplify the daily work of the cardiologists. The method may also improve accuracy of cardiac pathology detection by wearable devices^48^. The proposed P wave detector represents a huge step towards fully automated systems for ECG analysis and diagnosis of cardiac arrhythmias.
